# Biofilm Formation, Antibiotic Resistance, and Virulence Analysis of Human and Avian Origin *Klebsiella pneumoniae* from Jiangsu, China

**DOI:** 10.3390/vetsci12070628

**Published:** 2025-06-30

**Authors:** Yulu Xue, Fangyu Shi, Bangyue Zhou, Yi Shi, Wenqing Luo, Jing Zhu, Yang Yang, Sujuan Chen, Tao Qin, Daxin Peng, Yinyan Yin

**Affiliations:** 1 College of Public Health, College of Medicine, Yangzhou University, Yangzhou 225009, China; mx120221179@stu.yzu.edu.cn (Y.X.); mx120231219@stu.yzu.edu.cn (W.L.); mx120231147@stu.yzu.edu.cn (J.Z.); 2 Southeast University Affiliated Xuzhou Central Hospital, Xuzhou 221009, China; 19952199261@163.com (F.S.); sxbsy723x@petalmail.com (Y.S.); 3 Clinical Medical College, Yangzhou University, Northern Jiangsu People’s Hospital, Yangzhou 225091, China; abangnnn@163.com; 4 College of Veterinary Medicine, Yangzhou University, Yangzhou 225009, China; yy@yzu.edu.cn (Y.Y.); chensj@yzu.edu.cn (S.C.); 5 Jiangsu Co-Innovation Center for the Prevention and Control of Important Animal Infectious Disease and Zoonoses, Yangzhou University, Yangzhou 225009, China; 6 Guangling College, Yangzhou University, Yangzhou 225000, China; 7 International Research Laboratory of Prevention and Control of Important Animal Infectious Diseases and Zoonotic Diseases of Jiangsu Higher Education Institutions, Yangzhou University, Yangzhou 225009, China; 8 Jiangsu Key Laboratory of Zoonosis, Yangzhou University, Yangzhou 225009, China

**Keywords:** *Klebsiella pneumoniae*, avian-origin, human-origin, biofilm, antibiotic resistance, virulence

## Abstract

*Klebsiella pneumoniae*, a zoonotic pathogen of global concern, poses significant threats to both veterinary and public health. Here, a comparative study characterized 14 clinical isolates (7 avian-derived, 7 human-derived) from Jiangsu, China, through integrated genomic and phenotypic analyses. Firstly, multilocus sequence typing (MLST) revealed distinct epidemiological patterns: the same ST type in avian isolates was circulating between different species and different regions, whereas it was not found in human isolates. In addition, hypervirulent *Klebsiella pneumoniae* (hvKP) phenotypes confirmed by string test were exclusive to two human isolates (*KP*15, *KP*20). Secondly, biofilm detection demonstrated 78.6% (11/14) of isolates possessed biofilm-forming capacity, with cellulose but not curli as the predominant matrix component. Human-derived *KP*15 and *KP*20 had the strongest biofilm formation ability in all isolates. Antimicrobial susceptibility profiling identified serious multidrug resistance in both avian and human isolates. Virulence gene analysis revealed striking disparities, with human isolates harboring 10–20 virulence factors (median 15) versus 6–7 (median 6.5) in avian counterparts. Finally, functional pathogenesis assessments demonstrated human-derived strains exhibited stronger epithelial cell adhesion (2-fold higher) and invasion (1.97-fold higher) in Calu-3 cell models and paradoxically showed reduced macrophage phagocytosis (2.85-fold lower at 2 h) for immune escape. In vivo models confirmed dose-dependent mortality, with human isolates demonstrating higher lethality in both *Galleria mellonella* and mice. Virulence gene burden positively correlated with mortality outcomes. These findings delineate critical host adaptation differences in *Klebsiella pneumoniae* populations and provide empirical evidence for pathogen transmission dynamics at the human-animal interface.

## Simple Summary

*Klebsiella pneumoniae* can be spread between humans and animals through close contact with food and the environment, and there is a risk of transmission from animals to humans. However, the correlation between human and avian sources of *Klebsiella pneumoniae* is not clear. Firstly, *Klebsiella pneumoniae* strains origined from human/avian in Jiangsu province, China, were isolated and identified. Secondly, the pathogenicity was evaluated in vitro and in vivo. We found that both avian-origin and human-origin strains possessed the ability to form biofilms and exhibited multi-drug resistantance, but with different ST types distributions. Additionally, the number of virulence genes in human-origin strains was found to be higher than that in avian-origin strains, and a positive correlation was observed between the number of virulence genes and pathogenicity. This study provided a theoretical basis for the prevention and control of diseases associated with *Klebsiella pneumoniae* by revealing the difference of biological characteristics between avian and human sources.

## 1. Introduction

*Klebsiella pneumoniae* (*K. pneumoniae*) is one of the most important enterobacteria of the *Klebsiella* genus, and diseases caused by *K. pneumoniae* account for more than 95% of *Klebsiella* infections [1]. *K. pneumoniae* is common in the respiratory tract and digestive tract of humans and animals, as well as in the natural environment, such as water and soil [2]. *K. pneumoniae* is an important pathogen of community-acquired pneumonia and can cause a variety of human infections, including pneumonia, bloodstream infections, meningitis, urinary tract infections, and invasive diseases [3]. *K. pneumoniae* not only has a serious impact on human health but also causes huge economic losses to the aquaculture industry. In recent years, *K. pneumoniae* has caused great harm to the health of poultry, domestic animals, and wild animals all over the world, and its morbidity and mortality have increased significantly [4]. *K. pneumoniae* can infect animals with different clinical symptoms, such as mastitis, pneumonia, and septicemia infection in pigs [5]; mastitis and respiratory tract infection in cows [6]; pneumonia and upper respiratory tract infection in sheep and goats [7]; and pneumonia, enteritis, liver abscess, and septicemia in poultry [8]. According to the expression level of virulence factors, clinical infection characteristics, and molecular biological characteristics such as the carriage of virulence genes and capsular serotypes, *K. pneumoniae* can be divided into classical *K. pneumoniae* (cKP) and hypervirulent *K. pneumoniae* (hvKP) [9]. According to data provided by the China Antimicrobial Resistance Surveillance System (CARSS), the national Bacterial Resistance Detection Report for 2022 shows that *K. pneumoniae* ranks second in prevalence among isolated Gram-negative pathogens with 21.2%.

The pathogenicity of *K. pneumoniae* is jointly determined by a variety of virulence genes, including capsular polysaccharide (CPS), lipopolysaccharide (LPS), fimbriae, iron carrier, and biofilm [10]. Capsular polysaccharide is an acidic polysaccharide matrix wrapped on the surface of *K. pneumoniae*, which can protect the bacteria against harsh environments or harmful substances and from the influence of host immune response and is one of the main virulence factors of *K. pneumoniae* [11]. The synthesis of CPS is regulated by genes *rmpA*, *rmpA2*, and *magA* [12]. The synthesis of fimbriae is encoded by the *fim* and *mrk* gene cluster [13]. Iron is an indispensable metal element for bacterial growth and plays a crucial role in the infection process of *K. pneumoniae* [14]. The synthesis of iron carriers is regulated by genes *ureA*, *entB*, *iutA*, *iucA*, *iroB*, *ybtS*, *irp2*, *fyuA*, *aerobactin*, *kfu*, and *peg-344*. The bacteria in the biofilm are not easily killed by the host defense system, and the biofilm can promote the substance transfer of antibiotic resistance [15].

There have been a lot of studies on the epidemic transmission of *K. pneumoniae*, but there are few studies on the transmission between animals and humans, especially the transmission between avian and human. Alexis Dereeper et al. discovered human multidrug-resistant and high-risk *K. pneumoniae* in a pet hospital, indicating that STs are shared between pets and humans, and companion animals had the potential risk of transmitting *K. pneumoniae* to humans [16]. Sandra Pulss et al. found that bacterial sequence types and OXA-48 plasmids had significant overlap between humans and animals, reinforcing the idea of transmission between humans and animals [17]. In addition, Jun Sung Hong et al.’s study in South Korea also observed that the increase of CTX-M-55 in companion animals may affect antimicrobial resistance (AMR) transmission between humans and companion animals [18]. Fan Yang et al. found that *K. pneumoniae* strains from different hosts (including humans and animals) had common molecular types and similar phenotypes and speculated that these strains may be transmitted between humans and animals [19]. Thongpan Leangapichart et al. found pig-to-human transmission in a pig farm, indicating the possibility of zoonotic transmission in the *K. pneumoniae* clonal subpopulation [20]. Agricultural workers face a heightened occupational risk of exposure to livestock-associated *K. pneumoniae* and can become carriers, potentially transmitting it to close contacts like family members and the general public [21]. Studies have shown that the same sequence type of *K. pneumoniae* exists in a human blood specimen and a sample of retail chicken meat [22]. A study in Norway showed that *K. pneumoniae* from the cecum of broilers and turkeys belongs to the same sublineage as *K. pneumoniae* from human blood, urine, and feces [23]. Some research showed that there was a relatively higher presence of carbapenem-resistant *K. pneumoniae* among hospital inpatients (7.3%) compared to that in the meat products (2.7%) and farm animals (pig, 4.6%; chicken, 0.63%) in Qingdao, China [24]. To explore the correlation and differences in biological characteristics of *K. pneumoniae* from different host sources, *K. pneumoniae* from the avian and human were isolated in the Jiangsu region. We conducted comparative studies on the homology analysis, virulence factors, drug resistance, and pathogenicity to mammals of *K. pneumoniae* and analyzed the possibility of transmission of *K. pneumoniae* between avian and human. This study provided data reference for the epidemiological study of *K. pneumoniae*.

## 2. Materials and Methods

### 2.1. Ethics Statement

The animal studies were in accordance with the Laboratory Animal Welfare and Ethics guidelines of the Jiangsu Provincial Laboratory Animal Management Committee and have been approved by it (permission number: SYXK(SU)2022-0044).

### 2.2. Reagents

Defibrinated sheep blood was purchased from SenBeiJia Biological Technology (Nanjing, China). Tryptone and yeast extract were purchased from OXOID (Basingstoke, UK). NaCl was obtained from National Pharmaceutical Group (Shanghai, China). Agar was purchased from Sincere Biotech (Shanghai, China). 2 × FineTaq PCR SuperMix (+dye) was purchased from TransGen Biotech (Shanghai, China). DL1000 DNA marker and DL2000 DNA marker were purchased from Takara (Kyoto, Japan). Antimicrobial discs were derived from Microbial Reagent (Hangzhou, China). Crystal violet, Congo red, and Triton X-100 were purchased from Sangon Biotech (Shanghai, China). LB broth was purchased from Hope Biotechnology (Qingdao, China). Calofluor White Stain was purchased from Sigma-Aldrich (St. Louis, MO, USA). DMEM medium, fetal bovine serum (FBS), and penicillin-streptomycin were sourced from Thermo Fisher Scientific (Waltham, MA, USA).

### 2.3. Isolation and Identification of K. pneumoniae

*K. pneumoniae* were isolated from sputum, blood, liver, heart, and feces of Xuzhou Central Hospital, Northern Jiangsu People’s Hospital, and animals’ tissues of Jiangsu Province from 2020 to 2023 (Table 1). The samples in this study followed the principle of randomization. Each sample was streaked on blood agar and incubated for 12–18 h at 37 °C. A single colony exhibiting suspicious characteristics, including white coloration, mucus-like consistency, and rounded morphology, was selected and purified two times. The genomic DNA of bacterial isolates was extracted by the boiling method as a template. PCR detection was performed using universal bacterial primers to amplify the 16S rRNA gene of the isolated strains. PCR program: 4 min pre-denaturation at 94 °C; 30 s denaturation at 94 °C; 45 s annealing at 51 °C; 1 min extension at 72 °C, for a total of 30 cycles; 10 min extension at 72 °C. Use 1% agarose gel electrophoresis to validate the PCR products. The purified PCR products were sent to Nanjing Qingke Biotechnology Co., Ltd. (Nanjing, China) for sequencing. The assembled complete sequences were subjected to nucleic acid sequence alignment on NCBI. The primers were synthesized by Sangon Biotech Co., Ltd. (Shanghai, China), and the primer sequences are shown in Table S1. The bacterial liquid is mixed with sterile 50% glycerol in a 1:1 ratio and stored at −80 °C for further studies.

### 2.4. Multilocus Sequence Typing and Homology Analysis

Molecular typing of *K. pneumoniae* was performed using the multilocus sequence typing (MLST) method. The seven housekeeping genes of *K. pneumoniae* (*gapA*, *infB*, *mdh*, *phoE*, *pgi*, *rpoB*, and *tonB*) were amplified by PCR and sequenced. PCR program: 5 min pre-denaturation at 94 °C; 45 s denaturation at 94 °C; 30 s annealing at 50 °C; 1 min extension at 72 °C, for a total of 30 cycles; 5 min extension at 72 °C. PCR products were separated by agarose gel electrophoresis in a 1% (*w*/*v*) gel. The purified PCR products were sent to Nanjing Qingke Biotechnology Co., Ltd. (Nanjing, China) for sequencing. The alleles and STs were determined by matching the sequence from the database in the Pasteur Institute MLST website (http://www.pasteur.fr/en (accessed on 2 June 2025)). The complete sequences of corresponding ST types were downloaded from the database and uploaded to MEGA11. The phylogenetic tree was constructed by the neighbor-joining method to analyze the genetic relationship between *K. pneumoniae* with different ST types.

### 2.5. String Test and Mucoviscosity Assay

The strains were inoculated onto LB agar plates and incubated at 37 °C for 12–18 h. The bacterial colonies were elongated using a typical bacteriologic loop stretched a mucoviscous string from the colony. The positive result of the string test was defined as the formation of viscous strings ≥5 mm [25]. The mucoviscosity assay was performed with the previous method [26]. Both the string test and mucoviscosity test are used to detect the viscosity of *Klebsiella pneumoniae*.

### 2.6. Biofilm Formation Assay

To test the biofilm formation ability of *K. pneumoniae* isolates, we used test tubes as described previously [27]. Briefly, *K. pneumoniae* isolates were at 37 °C for 8–10 h. After the OD_600_ of the bacteria solution reached 0.8~1.1, the bacteria were inoculated in test tubes and then incubated for 24 h at 37 °C. Next, the test tubes were washed with PBS. Subsequently, 0.4% crystal violet (CV) solution was added to the test tubes and incubated at room temperature for 30 min away from light. Then, the test tubes were washed three times with PBS and allowed to dry. The CV was dissolved using absolute ethanol, and the absorbance was measured at a wavelength of 550 nm using a microplate reader. The experiment was independently repeated three times.

The biofilm formation ability of *K. pneumoniae* isolates was divided according to the ODc [28] (ODc = mean absorbance of negative controls + 3SD (the standard deviation)). OD_550_ ≤ ODc indicated that the strain was unable to form biofilms; ODc < OD_550_ ≤ 2ODc indicated that the strain had a weak ability to form biofilms. 2ODc < OD_550_ ≤ 4ODc indicated that the strain had moderate biofilm formation ability. OD_550_ > 4ODc indicated that the strain had a strong ability to form biofilms.

Curli and cellulose production were detected by Congo Red (CR) plates and Calcofluor (CF) plates [29]. 10 μL of bacterial solution was inoculated on Congo Red plates and Calcofluor plates and incubated at 28 °C for 96 h. Expression of curli fimbriae and cellulose nanofibers shows the dry, red, and rough morphotype on Congo red agar plates. Only expression of cellulose nanofibers shows the pink and smooth morphotype, while only expression of curli fimbriae has the brown, dry, and rough morphotype [30]. Colony morphology on the Calcofluor plate was observed under an Ultraviolet (UV) light at 365 nm [31]. The experiment was independently repeated three times.

### 2.7. Antibiotic Susceptibility Test

Antibiotic susceptibility testing was performed and interpreted using the standard Kirby–Bauer disk diffusion method according to CLSI (2020) [32]. In this study, a total of 20 antibiotics were tested. *Escherichia coli* ATCC25922 was used as a quality control strain for parallel testing to ensure the validity of the results.

### 2.8. Virulence Gene Detection

To determine the virulence profile of the *K. pneumoniae* isolates, 21 virulence genes were tested by PCR. These virulence genes include lipopolysaccharide-related genes (*uge* [33], *wabG* [34]), capsular polysaccharide synthesis and synthesis regulation-related genes (*rmpA* [35], *rmpA2* [36], *magA* [35], *K2* [35], *wcaG* [37]), fimbriae synthesis-related genes (*fimH* [34], *mrkD* [35]), urease-related gene (*ureA* [33]), allantoin-related gene (*allS* [35]), and iron uptake system genes (*entB* [35], *iutA* [35], *iucA* [36], *iroB* [36], *ybtS* [35], *irp2* [36], *fyuA* [38], *kfu* [35], *aerobactin* [39], *peg-344* [36]). Primers used in this study are listed in Table S1. The PCR program refers to the references of each virulence gene. PCR products were analyzed on a 1% agarose gel. The presence of a target gene was confirmed if amplicons were of the expected size.

### 2.9. Adhesion and Invasion Assay of K. pneumoniae to Calu-3 Cell

The adhesion and invasion of epithelial cells by *Klebsiella pneumoniae* are mainly based on relevant references [40]. Calu-3 cells were cultured in Dulbecco’s modified Eagle medium (DMEM) with 10% fetal bovine serum at 37 °C and 5% CO_2_, respectively. For adhesion assays, bacterial strains were added at an MOI of 10:1 bacteria/cell proportion and incubated for 1 h. Non-adherent bacteria were removed by washing 3 times with 1 mL sterile PBS. Then, cells were lysed with 0.5% TritonX-100 at 37 °C for 10 min. The cell lysates were serially diluted with PBS and cultured on LB plates to calculate the number of viable bacteria. For invasion assays, after 1 h of incubation with bacteria, 100 μg/mL gentamicin was added and incubated at 37 °C for 1 h. The cell lysates were cultured on LB plates after cell washing and lysing. The experiment was independently repeated three times.

### 2.10. Phagocytosis and Clearance Assay

To detect the ability of *K. pneumoniae* to resist phagocytosis and clearance of macrophages. RAW264.7 cells were cultured in Dulbecco’s modified Eagle medium (DMEM) with 10% fetal bovine serum at 37 °C and 5% CO_2_. For the phagocytosis assay, bacterial strains were added at an MOI of 100:1 bacteria/cell proportion and incubated for 1 h. The initial number of cells was 2.5 × 10^5^ per well in a 24-well plate. After incubation, the cells were washed with sterile PBS. 100 μg/mL gentamicin was added and incubated at 37 °C for 1 h to kill bacteria that did not enter cells. Then, the cells were washed with sterile PBS and lysed with 0.5% TritonX-100 at 37 °C for 10 min. The cell lysates were cultured on LB plates to calculate the number of viable bacteria. For the clearance assay, after 1 h of incubation with 100 μg/mL gentamicin, the cells were washed with sterile PBS, and 10 μg/mL gentamicin was added and incubated at 37 °C for 7 h and 20 h. The cell lysates were cultured on LB plates. The experiment was independently repeated three times.

### 2.11. Pathogenicity of K. pneumoniae in Galleria Mellonella and Mice

To evaluate the virulence of *K. pneumoniae* isolates, the *Galleria mellonella* infection model was established as described previously [41]. The larvae of the *Galleria mellonella* were fed at 37 °C for 24 h and then were randomly divided into seventy-three groups (*n* = 10 per group). The penultimate left foot of larvae in each group was injected with 20 μL of the bacterial suspension or sterile PBS. Survival rates of *Galleria mellonella* were recorded for 5 days. The lethal dose 50 (LD_50_) was calculated by the modified Koch’s method.

The pneumonia murine model was established as previously described [42]. Female SPF C57BL/6 mice aged 6–8 weeks were used in this study. Five mice were randomly assigned to each cage. Food and water can be freely obtained. Mice were weighed and anesthetized with an intraperitoneal injection of tribromoethanol. Then, each mouse was intratracheally injected with 20 μL bacterial suspension or sterile PBS. After the injection, the incision was glued with tissue adhesive. Weight change was assessed daily for the next 15 days, and the survival of the mice was recorded. The LD_50_ was calculated by a modified Koch’s method. In addition, in the group of *KP*820 and *KP*20, mice were euthanized at 24 h, 48 h, and 72 h after bacterial infection, and the heart, liver, spleen, and lung tissues were aseptically separated and weighed. Tissue weight/body weight ratio was calculated. Meanwhile, the heart, liver, spleen, and lung tissues were homogenized and plated onto LB plates to calculate the number of viable bacteria. The mice were euthanized with CO_2_.

### 2.12. Statistical Analysis

All the quantitative experiments were independently performed at least in triplicate. All data are expressed as mean and standard deviations (SD). The data were visualized using GraphPad Prism 9.5. ANOVA in SPSS 27.0 software was used to compare the statistical differences between different groups. The LSD method was used for multiple comparisons. * *p* < 0.05 indicated a statistically significant difference. Spearman’s correlation coefficient (r) was calculated to evaluate the correlation. |r| ≥ 0.8 indicated high correlation, 0.5 ≤ |r| < 0.8 indicated moderate correlation, 0.3 ≤ |r| < 0.5 indicated low correlation, and |r| < 0.3 indicated no correlation.

## 3. Results

### 3.1. Isolation and Identification of K. pneumoniae

In our study, a total of 14 *K. pneumoniae* strains were isolated from Jiangsu, China, during 2020 to 2023 (Table 1). Among these, seven strains of *K. pneumoniae* isolated from avian were named *KP*820, *KP*826, *KP*911, *KP*926, *KP*1016, *KP*1103, and *KP*1116, respectively. Seven strains of *K. pneumoniae* isolated from humans were named *KP*33, *KP*34, *KP*35, *KP*36, *KP*37, *KP*15, and *KP*20, respectively.

**Figure 1:**
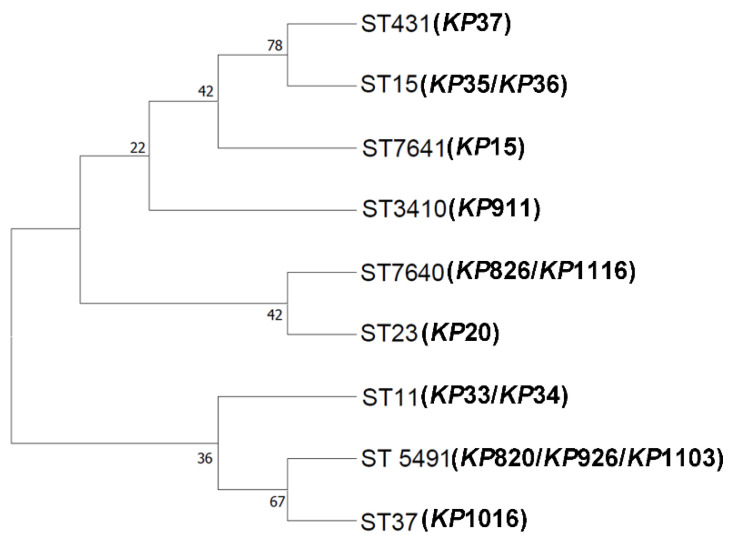
Neighbor-joining phylogeny was constructed using seven MLST genes for 14 *K. pneumoniae* isolates with 1000 bootstrap replicates. The high bootstrap values implied a high level of confidence in the tree.

MLST has been used for a variety of bacteria detection and evolutionary studies of epidemiology [43]. The technique was applied to the genotyping of *K. pneumoniae* in 2005 [44]. First, we examined the ST type of 14 *K. pneumoniae* isolates. As shown in Table 2, the MLST analysis revealed 9 different STs among 14 *K. pneumoniae*. *KP*826, *KP*1116, and *KP*15 were classified as two new STs (ST7640 and ST7641). ST37, ST3410, ST5491, and ST7640 were found in *K. pneumoniae* isolated from avian. *KP*820, *KP*926, and *KP*1103 belong to ST5491. *KP*826 and *KP*1116 belong to ST7640. ST11, ST15, ST23, ST431, and ST7641 were found in *K. pneumoniae* isolated from humans. *KP*33 and *KP*34 belong to ST11. *KP*35 and *KP*36 belong to ST15. Next, we conducted phylogenetic analyses to investigate the evolutionary distances between these isolates. As shown in Figure 1, *KP*820, *KP*926, *KP*1103, and *KP*1016 showed a high homology. *KP*35, *KP*36, and *KP*37 showed a high homology. *KP*20, *KP*826, and *KP*1116 also showed a high homology.

**Figure 2:**
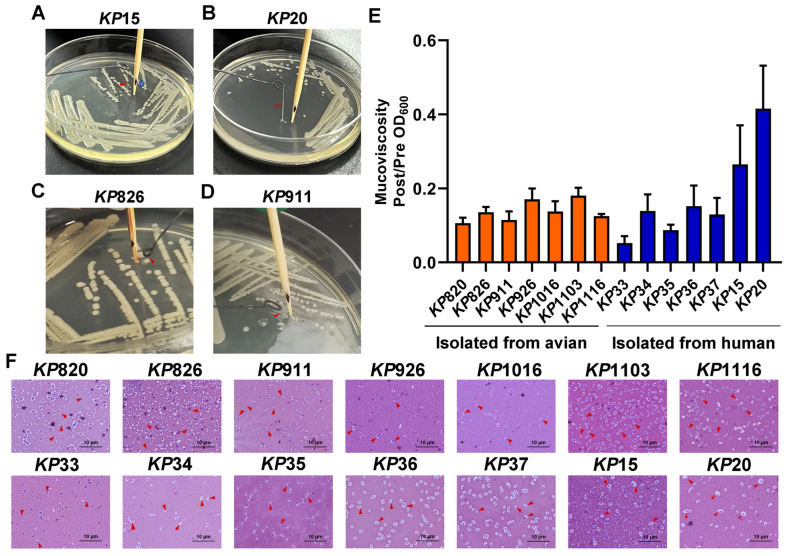
Results of viscosity assay. (**A**,**B**) *KP*15 and *KP*20 could form a string on the LB agar plate. Generally, a string 5 mm or longer was defined as positive. *, the distance from the bottom of the toothpick to the black spot is 5 mm. (**C**,**D**) *KP*826 and *KP*911 could not form a string on the LB agar plate. (**E**) Mucoviscosity assay of *K. pneumoniae* isolates. (**F**) The bacteria were evenly mixed on the slide with Congo red and serum mixture solution and then were pushed into a thin layer. The bacteria were decolonized with 5% dilute hydrochloric acid for 1 min, washed with water, and then stained with 0.1% crystal violet for 1 min, washed with water, dried naturally, and observed under the oil microscope. Bars: 10 μm. Capsules are indicated by short red arrows.

String testing is commonly used to determine the hypermucoviscosity (hmv) phenotype of *K. pneumoniae*, which is typical of most hvKP [45]. The evaluation of mucoviscosity can also be used to distinguish between hvKP and cKP strains [46]. Here, we conducted a string test and mucoviscosity assay for *K. pneumoniae*. As shown in Figure 2 and Table 3, only *KP*15 and *KP*20 from human *K. pneumoniae* could form a long string, while other strains did not have the ability. In addition, mucoviscosity was also detected. As shown in Figure 2E, *KP*15 and *KP*20, isolated from humans, had a higher mucoviscosity than other strains, which was consistent with the results of the string test. *KP*15 and *KP*20 were hvKP, while the remaining strains were cKP. Besides, the capsules of *K. pneumoniae* were stained with the Congo red negative staining method according to the reference [47]. As shown in Figure 2F, *KP*20 had the thickest capsule among the 14 strains of *K. pneumoniae*.

### 3.2. Biofilm Formation Ability of K. pneumoniae Strains

**Figure 3:**
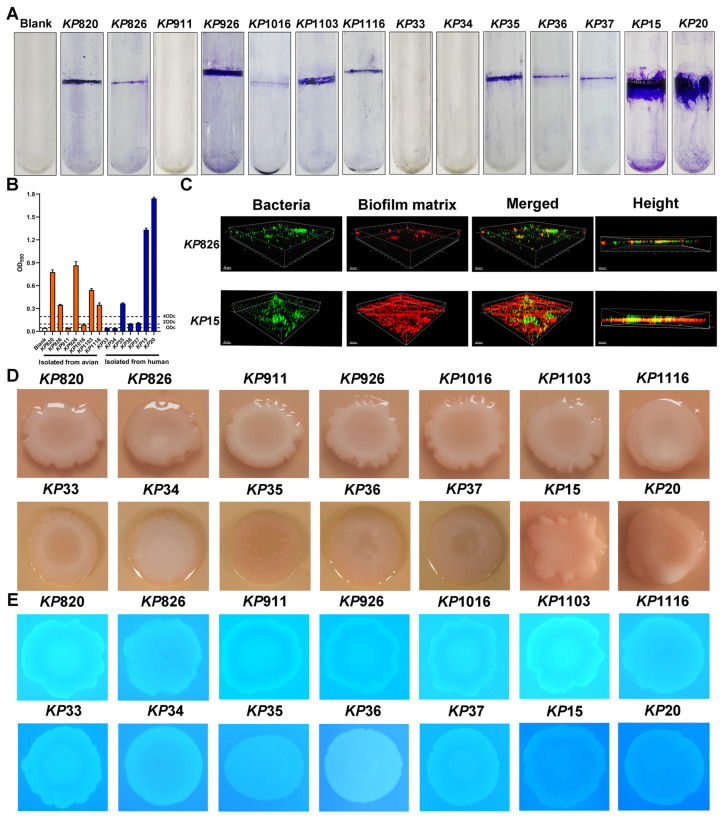
Results of biofilm formation ability of *K. pneumoniae* isolates. (**A**) The biofilm formation was detected by the crystal violet in the test tube. (**B**) The crystal violet was dissolved using absolute ethanol, and the absorbance was measured at a wavelength of 550 nm. (**C**) Biofilm structure of *KP*826 and *KP*15 was observed by confocal laser scanning microscopy (CLSM). (**D**) Colony morphology of 14 *K. pneumoniae* isolates growing on Congo red plates. (**E**) Colony morphology of 14 *K. pneumoniae* isolates growing on Calcofluor plates. Photographs represent one of three experiments, which gave similar results.

Biofilm formation is one of the major virulence properties of *K. pneumoniae* [48]. We evaluated the biofilm formation ability of *K. pneumoniae* isolates by using test tubes. As shown in Figure 3A, it is stated that 11 strains formed biofilms, with *KP*820/*KP*926 being the most potent in avian species and *KP*15/*KP*20 in humans. Next, the OD value was statistically analyzed, and biofilm formation ability was classified according to the OD value. As shown in Figure 3B, *KP*820, *KP*926, *KP*1103, *KP*1116, *KP*35, *KP*15, and *KP*20 had a strong biofilm formation ability; *KP*36 and *KP*37 had a moderate biofilm formation ability; *KP*1016 had a weak biofilm formation ability; and *KP*911, *KP*33, and *KP*34 had no biofilm formation ability. These data suggested that the majority of *K. pneumoniae* can form the biofilm. Meanwhile, biofilm structure of *KP*826 and *KP*15 was observed by confocal laser scanning microscopy (CLSM) in Figure 3C. In the process of biofilm formation, Alexa Fluor^TM^ 647 dextran was added, the biofilm matrix showed red fluorescence, and bacterial nucleic acids were stained with Syto 9 (green fluorescence). Compared with *KP*826, *KP*15 had a higher ability to form biofilms. Furthermore, we evaluated the curli and cellulose production of the *K. pneumoniae* strains on CR and CF agar plates. As shown in Figure 3D,E, 14 *K. pneumoniae* strains produced a pink colony, indicating a lack of curli production. We found that *KP*820 and *KP*1103 displayed strong fluorescence under UV light illumination, indicating their strong cellulose production capacity.

### 3.3. Antibiotic Susceptibility Patterns and Virulence Factors Distribution of K. pneumoniae Isolates

The antimicrobial resistance results are presented in Table 4 and Table 5. Among the strains isolated from avian, the resistance rate to cotrimoxazole, florfenicol, chloramphenicol, ampicillin, carbenicillin, erythromycin, tetracycline, doxycycline, nitrofurantoin, and rifampin was 100%. The resistance rate to ciprofloxacin, norfloxacin, cephalexin, cefotaxime, amikacin, and aztreonam was 71.43%. The resistance rate to cefoxitin was 57.14%. The resistance rate to meropenem, imipenem, and polymyxin B was 0%. *KP*820, *KP*926, *KP*1016, and *KP*1103 were resistant to 17 antibiotics. *KP*911 was resistant to 16 antibiotics. *KP*826 and *KP*1116 were resistant to 10 antibiotics. Among the strains isolated from humans, the resistance rate to cephalexin, ampicillin, carbenicillin, erythromycin, tetracycline, doxycycline, nitrofurantoin, and rifampin was 100%. The resistance rate to ciprofloxacin, norfloxacin, and aztreonam was 85.71%. The resistance rate to cotrimoxazole, cefotaxime, amikacin, and imipenem was 71.43%. The resistance rate to cefoxitin and meropenem was 57.14%. The resistance rate to chloramphenicol and polymyxin B was 28.57%. The resistance rate to florfenicol was 14.29%. *KP*34 and *KP*35 were resistant to 18 antibiotics. *KP*33 was resistant to 17 antibiotics. *KP*36 was resistant to 16 antibiotics. *KP*37 and *KP*15 were resistant to 15 antibiotics. *KP*20 was resistant to 8 antibiotics. We found that all *K. pneumoniae* were completely resistant to ampicillin, carbenicillin, erythromycin, tetracycline, doxycycline, nitrofurantoin, and rifampin. It was noted that the isolates in this study were all multi-drug resistant (MDR) strains, indicating that the resistance status of *K. pneumoniae* is severe.

To further investigate the molecular characteristics of these *K. pneumoniae* strains, we analyzed their virulence gene profiles. As shown in Table 6 and Table 7, 21 virulence genes in this study were detected. All *K. pneumoniae* strains carried *uge*, *wabG*, *fimH*, *mrkD*, *ureA*, and *entB*. All strains isolated from humans carried *ybtS*, *irp2*, and *fyuA*. All strains isolated from avian did not carry *ybtS*, *irp2*, and *fyuA*. *RmpA*, *rmpA2*, *magA*, *K2*, *wcaG*, *allS*, *iutA*, *iucA*, *iroB*, *aerobactin*, and *peg-344* can be found in some strains isolated from humans, but not in strains isolated from avian. The *kfu* gene was found in *KP*820, *KP*926, *KP*1103, *KP*35, *KP*36, *KP*37, and *KP*20. All *K. pneumoniae* isolates had three or more virulence genes, and the number of virulence genes of *KP*20 reached 20. The virulence genes of *K. pneumoniae* isolated from avian and human sources were 6–7 and 10–20, respectively. We conducted a statistical analysis on the difference in the number of virulence genes carried by avian and human *K. pneumoniae*. As shown in Figure S1. The number of virulence genes carried by human strains is significantly higher than that of avian strains. Besides, we found no differences in the virulence genes carried by strains of the same ST except for ST11. These data suggested that the virulence of *K. pneumoniae* isolated from humans may be stronger than *K. pneumoniae* isolated from avian.

### 3.4. Adhesion and Invasion of K. pneumoniae to Calu-3 Cells

**Figure 4:**
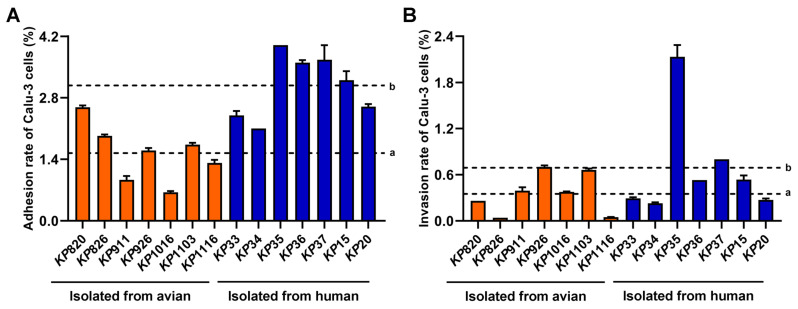
Result of epithelial cell adhesion and invasion assay. (**A**) Adhesion ability of *K. pneumoniae* to Calu-3 cells. (**B**) Invasion ability of *K. pneumoniae* to Calu-3 cells. a: The average adhesion rate or invasion rate of strains isolated from avian to Calu-3 cells. b: The average adhesion rate or invasion rate of strains isolated from humans to Calu-3 cells.

To evaluate the adhesive and invasive capacity of *K. pneumoniae*, strains infected Calu-3 cells. As shown in Figure 4A,B, the average adhesion (a = 1.54%, b = 3.08%) and invasion rate (a = 0.35%, b = 0.69%) of strains isolated from humans to Calu-3 cells were higher than that of strains isolated from avian. Among the strains isolated from avian, *KP*820 showed the highest adhesion rate to Calu-3 cells. *KP*926 showed the highest invasion rate to Calu-3 cells. Among the strains isolated from humans, *KP*35 showed the highest adhesion rate and invasion rate to Calu-3 cells. Statistical analysis was conducted on the differences in adhesion and invasion of Calu-3 cells between avian strains and human strains. As shown in Figure S2. The adhesion rate and invasion rate of human strains to Calu-3 cells were significantly higher than those of avian strains. These data suggested that the adhesion and invasion ability of strains isolated from humans to Calu-3 cells was stronger than that of strains isolated from avian.

### 3.5. Phagocytosis of K. pneumoniae by Macrophages and Proliferation of K. pneumoniae in Macrophages

**Figure 5:**
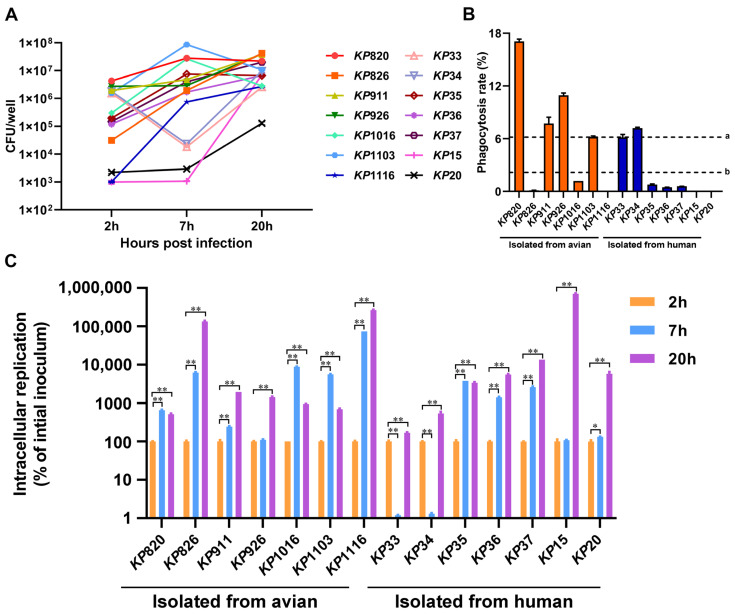
Results of *K. pneumoniae* infection in macrophage RAW264.7. (**A**) The amount of *K. pneumoniae* strains inside macrophage RAW264.7 at 2, 7, and 20 h after infection, respectively. (**B**) Phagocytosis rate of macrophages RAW264.7 against *K. pneumoniae*. a: The average phagocytosis rate of macrophages to strains isolated from avian. b: The average phagocytosis rate of macrophages to strains isolated from humans. (**C**) The intracellular replication rates of *K. pneumoniae* strains inside macrophage RAW264.7. The intracellular replication at 7 and 20 h was calculated by comparing to the CFUs at 2 h *, *p* < 0.05; **, *p* < 0.01.

We evaluated the phagocytic ability of macrophages to *K. pneumoniae*. As shown in Figure 5B, the phagocytosis rate of macrophages to strains isolated from avian was higher than that of strains isolated from human (a = 6.18%, b = 2.17%) at 2 h. Among the strains isolated from avian, macrophages had the strongest phagocytic ability to *KP*820. Among the strains isolated from humans, macrophages had the strongest phagocytic ability to *KP*34. Statistical analysis was conducted on the differences in macrophage phagocytosis between avian strains and human strains. As shown in Figure S3. The phagocytic rate of avian strains by macrophages was significantly higher than that of human strains. Next, we counted the number of *K. pneumoniae* in macrophages after infection with *K. pneumoniae* at 7 and 20 h, respectively. As shown in Figure 5A,C, compared to that at 2 h, the intracellular replication of *K. pneumoniae* was significantly increased at 20 h (*p* < 0.05). Among the strains isolated from avian, *KP*1116 had the strongest intracellular replication ability in macrophages. Among the strains isolated from humans, *KP*15 had the strongest intracellular replication ability in macrophages.

### 3.6. The Results of K. pneumoniae Infection in Galleria Mellonella and Mice

**Figure 6:**
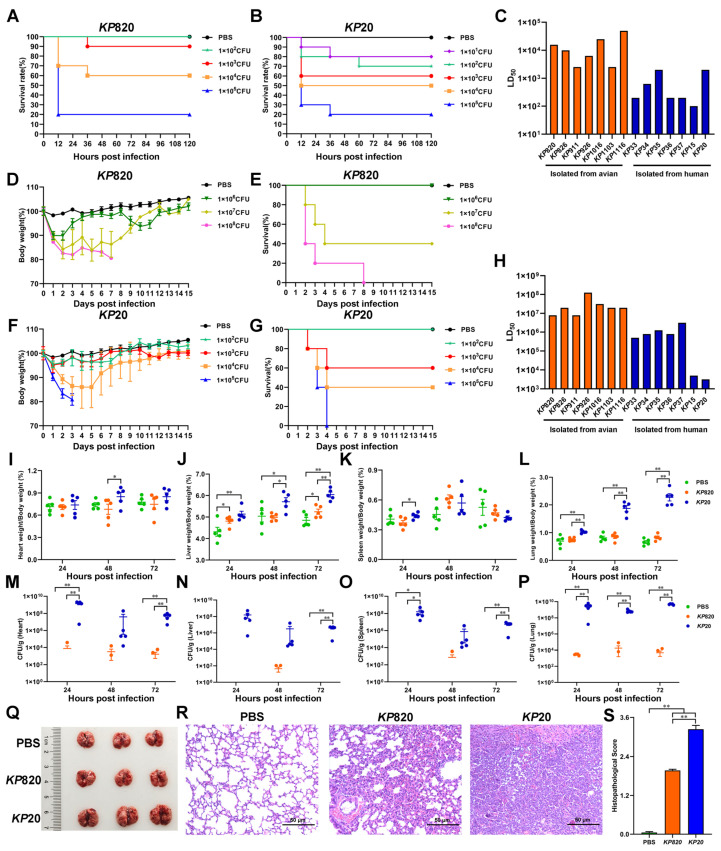
Evaluation of pathogenicity of *K. pneumoniae* isolates. (**A**) Survival rate of *Galleria mellonella (n* = 10) infected with *KP*820. (**B**) Survival rate of *Galleria mellonella* (*n* = 10) infected with *KP*20. (**C**) *K. pneumoniae* strains infected *Galleria mellonella*, then calculated median lethal dose. (**D**,**E**) The body weight changes and survival rate of mice (*n* = 5) intratracheally injected with *KP*820. (**F**,**G**) The body weight changes and survival rate of mice (*n* = 5) intratracheally injected with *KP*20. (**H**) *K. pneumoniae* strains infected mice, then calculated the median lethal dose. (**I**–**L**) The wet weight of the tissue (heart, liver, spleen, and lung) in mice (*n* = 5) intratracheally injected with PBS, *KP*820, and *KP*20 at 24, 48, and 72 h post infection. (**M**–**P**) The bacterial load of the tissue (heart, liver, spleen, and lung) in mice (*n* = 5) intratracheally injected with PBS, *KP*820, and *KP*20 at 24, 48, and 72 h post infection. The pathology images in lungs (*n* = 3 per group) (**Q**), histopathological images in H&E-stained lung tissues (*n* = 3) (**R**), and histopathologic scores in the lungs (**S**) of mice intratracheally injected with PBS, *KP*820, and *KP*20 at 72 h post infection. *, *p* < 0.05; **, *p* < 0.01.

The *Galleria mellonella* infection model has been employed to investigate various bacteria and assess the virulence of *K. pneumoniae* due to the ease and cost-effectiveness of obtaining larvae [49]. In this study, we counted the survival rate of *Galleria mellonella* infected with *K. pneumoniae* for 5 days and calculated the LD_50_ by modified Koch’s method. As shown in Figure 6A–C and Figure S4, the LD_50_ of strains isolated from avian was above 2.5 × 10^3^, and the LD_50_ of strains isolated from human was below 2 × 10^3^. Among the strains isolated from avian, *KP*911 and *KP*1103 were the most virulent to *Galleria mellonella*. Among the strains isolated from humans, *KP*15 was the most virulent to *Galleria mellonella*.

Mouse infection models remain a standard for assessing pathogen virulence, including that for *K. pneumoniae*, which is an accurate method for identifying hvKP and differentiating it from cKP [45]. In this study, we evaluated the body weight, survival rate, and median lethal dose of mice intratracheally injected with *K. pneumoniae*. The results are shown in Figure 6D–H, Figures S5 and S6. The LD_50_ of strains isolated from avian was above 8 × 10^6^, and the LD_50_ of strains isolated from humans was below 3.2 × 10^6^. Among the strains isolated from avian, *KP*820 and *KP*911 were the most virulent to mice. Among the strains isolated from humans, *KP*20 was the most virulent to mice. In addition, the correlation between the number of virulence genes and the LD_50_ of the *Galleria mellonella* and mice was analyzed. As shown in Table 8. The number of virulence genes was moderately negatively correlated with the LD_50_ of *Galleria mellonella* and highly negatively correlated with the LD_50_ of mice.

According to the results of LD_50_ to mice, *KP*820 and *KP*20 were the most virulent strains isolated from avian and human, respectively. Next, we measured the tissues’ wet weight and bacterial load of mice infected with PBS, *KP*820, and *KP*20. As shown in Figure 6I–L, compared to the PBS group, the wet liver weights were significantly (*p* < 0.05) increased in the *KP*820 group after 24 and 72 h post infection. Compared to the *KP*820 group, the wet liver weights and wet lung weights were significantly (*p* < 0.05) increased in the *KP*20 group after 48 and 72 h post infection. As shown in Figure 6M–P, compared to the PBS group, the bacterial loads of the heart, liver, spleen, and lung were significantly (*p* < 0.01) increased in the *KP*20 group after 72 h post infection. Compared to the *KP*820 group, the bacterial loads of the lung were significantly (*p* < 0.01) increased in the *KP*20 group after 24, 48, and 72 h post infection. At 72 h post infection, the lungs of challenged mice in the *KP*20 group showed swelling (Figure 6Q) and severe pneumonia with inflammatory cellular infiltration and alveolar wall thickening by an H&E staining (Figure 6R). Compared to the *KP*20 group, the pathological changes in the lungs of mice in the *KP*820 group were milder, and the pathological index was lower (Figure 6S).

## 4. Discussion

*K. pneumoniae* is a zoonotic pathogen widely distributed in nature, which can cause respiratory tract and urethral infections, meningitis, enteritis, and other tissue and organ inflammation and septicemia in humans and animals [50]. *K. pneumoniae* has caused economic losses to the breeding industry and posed a long-term threat to public health security and human health [51]. In this study, a total of 14 strains of *K. pneumoniae* were isolated from avian-origin samples and human hospitals, respectively, and their biological characteristics were compared. We found that the same ST type in *K. pneumoniae* isolated from avian was prevalent between different species and different regions. Most of *K. pneumoniae* isolated from avian and human sources could form biofilms, and the biofilms were mainly composed of cellulose and did not contain culri. *K. pneumoniae* isolated from avian and human sources are multi-drug resistant strains, but there were different drug resistance profiles, which were related to the use of specific antibiotics. *K. pneumoniae* isolated from avian had certain pathogenicity to Galleria mellonella and mice; *K. pneumoniae* isolated from humans had stronger pathogenicity.

The phylogenetic tree showed the relationship between the ST types of different strains. Generally, strains belonging to the same branch were closely related. There are 9 ST types of *K. pneumoniae* in this study, among which ST7640 and ST7641 are newly discovered ST types. Notably, ST5491 was found in chickens, ducks, and Grus Japonensis, and ST7640 was found in ducks and Grus Japonensis, indicating possible cross-species transmission of *K. pneumoniae*. ST11, ST15, and ST431 were prevalent in Xuzhou. ST5491 was found in Dongtai, Jiangyin, and Sheyang, indicating that ST5491 may be transmitted across regions. Although this study did not detect the same ST type in animals and humans, ST7640 and ST23 belong to the same clade, indicating that there may be mutual transmission between animals and humans. The results of this study were consistent with a study in Qingdao. There was no overlap in STs between the isolates from hospitalized patients and those from meat products or farm animals [24]. More studies are needed to confirm the possibility of transmission of *K. pneumoniae* between animals and humans. This study confirmed that *K. pneumoniae* has cross-species and cross-regional transmission, which is not conducive to the prevention and control of *K. pneumoniae*, and strict infection prevention and control measures should be implemented to reduce the risk of transmission and infection.

*K. pneumoniae* can form biofilms, which can enhance resistance to the attack of the host immune system [52]. The existence of the biofilm is also closely related to its virulence and drug resistance [53]. In this study, the results of the test tube showed that 78.6% of *K. pneumoniae* formed biofilms. Sanchez et al. have shown that MDR strains had a high positive rate of biofilm formation, and MDR *K. pneumoniae* that can form biofilms may cause the infection to become difficult to treat [53]. Curli and cellulose are the primary extracellular components that play crucial roles in the adhesion of bacteria to surfaces and the formation of biofilms [54]. In this study, 14 *K. pneumoniae* strains lacked curli in biofilms. *KP*820 and *KP*1103 contained a higher cellulose production. Studies have shown that cellulose is an important protective scaffold for biofilm formation, and the increase of its synthesis can enhance the stability of biofilm [55].

In recent years, the resistance rate of *K. pneumoniae* has gradually increased, and more and more MDR strains have appeared [56]. MDR is a key problem in the control of *K. pneumoniae* [57]. The 14 strains of *K. pneumoniae* in this study were all multidrug-resistant strains. Multiple drug resistance and high virulence have long been considered two different evolutionary directions, and hvKP is usually sensitive to antibiotics [58]. *KP*20 in this study fits this profile. Avian strains were completely sensitive to meropenem, imipenem, and polymyxin B, which may be because carbapenems and polymyxins are not approved for feeding food animals [59]. Human strains had a relatively high resistance rate to imipenem and meropenem. Research showed that the resistance rates of *K. pneumoniae* to imipenem and meropenem increased from 7.8% and 9.6% in 2019 to 11.6% and 13.2% in 2023, respectively, much higher than the resistance rates of *E. coli* to carbapenem antibiotics (<2%) [60]. It indicated that the current situation of drug resistance of clinical patients to carbapenem antibiotics is becoming increasingly serious. Sulfonamide antibiotics had been used as feed additives to improve the growth and yield of livestock and poultry food animals [33], which was consistent with the complete resistance of *K. pneumoniae* isolated from avian to sulfonamide antibiotics in this study. Among the *K. pneumoniae* isolated from avian, two strains came from the feces of Grus Japonensis. Studies have shown that contaminated feed, soil, and surface water can spread antibiotic resistance [61], that feces can become a reservoir of antibiotic compounds and resistant bacteria [62], and that birds become vectors for the spread of resistant bacteria across geographic distances [63]. *K. pneumoniae* can be transmitted to humans by infecting livestock and poultry or contaminating retail meat [64]. Resistant bacteria can be enriched by selection and return to human patients via direct contact, the environment, or the food chain [65]. Therefore, strict prevention and control measures should be implemented to prevent the transmission of *K. pneumoniae* between humans and avian, such as regular cleaning and disinfection of farms, improving sanitation, and minimizing contact with livestock and poultry.

Virulence factors play important roles in different stages of pathogen infection and pathogenicity [66]. Compared with cKP, hvKP produces more iron carriers with stronger activity [67]. A study found that the virulence of *K. pneumoniae* may mainly depend on the number of virulence factors it contains [10]. In our study, there was no difference in the distribution of lipopolysaccharide virulence genes *uge* and *wabG*, *fimH* and *mrkD*, allantoin virulence gene *ureA*, and iron carrier virulence gene *entB* in *K. pneumoniae* isolated from avian and human sources. However, capsule virulence genes *rmpA*, *rmpA2*, *magA*, *K2*, *wcaG*, and other siderophore virulence genes except kfu were only present in some strains isolated from humans, but not in strains isolated from birds. Similar to a study in Faisalabad, Pakistan, none of the six strains of *K. pneumoniae* isolated from broilers carried *rmpA*, *rmpA2*, *magA*, *iucA*, *iroB,* and *irp2*, but all carried *fimH*, *mrkD*, *ureA* and *entB* [68]. Therefore, it is speculated that capsule virulence genes and siderophore virulence genes are the key factors for determining the difference in virulence between *K. pneumoniae* isolated from avian and humans.

*K. pneumoniae* can naturally exist in the gut and respiratory tract of healthy individuals, and when the body’s resistance is reduced, it can invade epithelial cells by adhesion or break the tight connections between epithelial cells, cross the epithelial cell barrier, and infect the body [69]. Our results showed that the adhesion and invasion ability of different *K. pneumoniae* to epithelial cells was different. *KP*35 has the strongest adhesion and invasion ability to epithelial cells.

After *K. pneumoniae* crosses the epithelial barrier, macrophages are involved in mediating congenital and acquired immune responses to reduce the harm to the body by phagocytosis to eliminate *K. pneumoniae* [70]. The ability to replicate within the macrophage is associated with virulence [71]. In this study, it was observed that macrophages had weak phagocytic capacity for *KP*826, *KP*1016, *KP*1116, *KP*35, *KP*36, *KP*37, *KP*15, and *KP*20. *K. pneumoniae* proliferated abundantly in macrophages after 20 h, indicating that *K. pneumoniae* could resist macrophage clearance capacity. In addition, the string test indicated that *KP*15 and *KP*20 were hvKP. Determination of virulence genes revealed that *KP*15 carried the capsular virulence gene *K2*, and *KP*20 carried the capsular virulence genes *magA* and *wcaG*. Meanwhile, *KP*15 and *KP*20 had a strong ability to resist phagocytosis by macrophages, and *KP*15 had the strongest intracellular replication ability in macrophages. Therefore, the ability of *K. pneumoniae* to resist phagocytosis by macrophages and its intracellular replication ability in macrophages may be related to the capsular structure. Similarly, Wanford, Joseph J., et al. found that hvKP resisted phagocyte-mediated clearance and replicated in mouse liver macrophages [72]. Combined with the results of the invasion assay, it can be found that *KP*35 has a strong ability to adhere to and invade Calu-3 cells and resist phagocytosis of macrophages. However, *KP*35 was not hvKP; it was speculated that this situation may be related to other pathogenicity of *K. pneumoniae*.

The virulence of both *K. pneumoniae* isolated from avian and human sources towards *Galleria mellonella* and mice was approximately identical. The virulence of *K. pneumoniae* isolated from humans was stronger than that of *K. pneumoniae* isolated from avian, and this finding was in line with the results of the virulence gene tests. According to the study results of Yee-Huang Ku et al., the carriage rate of virulence factors in isolates with LD_50_ < 10^6^ (high virulence) was significantly higher than in isolates with LD_50_ > 10^6^ (low virulence) [73]. In this study, we found that the number of virulence genes was positively correlated with the pathogenicity of mice and *Galleria mellonella.* It was speculated that the more virulence genes there are, the stronger the pathogenicity to mammals. In this study, the LD_50_ of the hvKP strain was about 10^3^, which was consistent with the results of Travis J. Kochan et al. [74]. The wet weight in the liver and lung of mice infected with *K. pneumoniae* isolated from humans was much higher than that of mice infected with *K. pneumoniae* isolated from avian. The bacterial load in the heart, liver, spleen, and lung of mice infected with *K. pneumoniae* isolated from humans was much higher than that of mice infected with *K. pneumoniae* isolated from avian. The wet weight and bacterial load in the lungs of mice infected with strains isolated from humans were about 2 to 3 times those of those infected with strains isolated from avian. Studies have shown that the lung is an organ directly connected with the external environment, which increases the chance of lung infection. The alveolar structure of the lung is also conducive to the adhesion and colonization of bacteria [75]. The liver is an organ with abundant blood circulation and is also vulnerable to bacterial invasion [76].

At present, existing studies have shown that plasmid-mediated horizontal gene transfer is an important factor for the widespread of bacteria within and between species. For example, several intestinal bacteria, including *K. pneumoniae,* have the ability to acquire and retransfer a broad-host-range plasmid, RP4. The RP4 plasmid can transfer to multiple clinically relevant bacterial species without antibiotic selection pressure [77]. The *Klebsiella* genus is highly mature in the microbiota of wild animals, making them a perfect reservoir for bacteria and the subsequent gene exchange [78]. *K. pneumoniae*, due to its outstanding ability to integrate β-lactamase genes, can efficiently insert these drug-resistant genes into mobile plasmids. This unique genetic plasticity, combined with the inherent horizontal transfer characteristics of the plasmid itself, directly promotes the evolution and global spread of plasmid-mediated cephalosporin enzymes in this strain [79].

This study had some limitations, such as a small sample size, the main sample sources being from the Jiangsu region, and the lack of complete genomic analysis. In the future, we will expand the sample size, extend the sampling range, and use whole-genome sequencing to strengthen the hypothesis of the transmission of *K. pneumoniae* between avian and human.

## 5. Conclusions

In summary, our study showed that different strains of avian origin had the same ST type, and this phenomenon existed in human strains. The same ST type existed in different regions and among different species. In addition, we found that the multiple drug resistance of *K. pneumoniae* isolated from avian and human sources was relatively serious. It was also found that *K. pneumoniae* strains isolated from humans were more pathogenic to mammals than *K. pneumoniae* strains isolated from avian. Therefore, it is more important to monitor the epidemiological spread of *K. pneumoniae* further and strengthen the management of its prevention and control to prevent it from causing more serious consequences.

## Figures and Tables

**Table 1:** The distribution and origin of *K. pneumoniae* isolates.

Strain	Host	Sample Type	Sample Source in Jiangsu
*KP*820	Chicken	Liver	Dongtai
*KP*826	Grus japonensis	Feces	Sheyang
*KP*911	Duck	Liver	Jiangyin
*KP*926	Duck	Heart	Jiangyin
*KP*1016	Duck	Liver	Yancheng
*KP*1103	Grus japonensis	Feces	Sheyang
*KP*1116	Duck	Liver	Jiangyin
*KP*33	Human	Sputum	Xuzhou
*KP*34	Human	Sputum	Xuzhou
*KP*35	Human	Sputum	Xuzhou
*KP*36	Human	Blood	Xuzhou
*KP*37	Human	Blood	Xuzhou
*KP*15	Human	Sputum	Yangzhou
*KP*20	Human	Sputum	Yangzhou

**Table 2:** Allele profile and ST typing of *K. pneumoniae* strains.

Strain	Allele Profile							ST
	*rpoB*	*gapA*	*mdh*	*pgi*	*phoE*	*infB*	*tonB*	
*KP*820	1	1	1	1	1	1	16	5491
*KP*826	18	17	92	84	122	19	162	7640 *
*KP*911	4	4	1	1	7	4	65	3410
*KP*926	1	1	1	1	1	1	16	5491
*KP*1016	1	2	2	1	13	9	16	37
*KP*1103	1	1	1	1	1	1	16	5491
*KP*1116	18	17	92	84	122	19	162	7640 *
*KP*33	1	3	1	1	1	3	4	11
*KP*34	1	3	1	1	1	3	4	11
*KP*35	1	1	1	1	1	1	1	15
*KP*36	1	1	1	1	1	1	1	15
*KP*37	1	2	1	1	1	1	1	431
*KP*15	4	1	500	1	10	1	13	7641 *
*KP*20	4	2	1	1	9	1	12	23

* New type.

**Table 3:** The results of the string test.

Strain	Result
*KP*820	−
*KP*826	−
*KP*911	−
*KP*926	−
*KP*1016	−
*KP*1103	−
*KP*1116	−
*KP*33	−
*KP*34	−
*KP*35	−
*KP*36	−
*KP*37	−
*KP*15	+
*KP*20	+

+ Positive. − Negative.

**Table 4:** Antimicrobial susceptibility profile of *K. pneumoniae* isolated from avian.

Antimicrobial Agents	Strain							Resistance Rate (%)
	*KP*820	*KP*826	*KP*911	*KP*926	*KP*1016	*KP*1103	*KP*1116	
SXT	R	R	R	R	R	R	R	100
FFC	R	R	R	R	R	R	R	100
C	R	R	R	R	R	R	R	100
CIP	R	I	R	R	R	R	I	71.43
NOR	R	I	R	R	R	R	I	71.43
CL	R	S	R	R	R	R	I	71.43
CTX	R	S	R	R	R	R	S	71.43
FOX	R	S	I	R	R	R	S	57.14
AK	R	S	R	R	R	R	I	71.43
AMP	R	R	R	R	R	R	R	100
CAR	R	R	R	R	R	R	R	100
E	R	R	R	R	R	R	R	100
TE	R	R	R	R	R	R	R	100
DO	R	R	R	R	R	R	R	100
F	R	R	R	R	R	R	R	100
ATM	R	S	R	R	R	R	S	71.43
MEM	S	S	S	S	S	S	S	0
IPM	S	S	S	S	S	S	S	0
PB	I	S	I	I	I	I	S	0
RD	R	R	R	R	R	R	R	100
Amount	17	10	16	17	17	17	10	

**Table 5:** Antimicrobial susceptibility profile of *K. pneumoniae* isolated from humans.

Antimicrobial Agents	Strain							Resistance Rate (%)
	*KP*33	*KP*34	*KP*35	*KP*36	*KP*37	*KP*15	*KP*20	
SXT	S	R	R	R	R	R	S	71.43
FFC	S	S	I	I	S	R	S	14.29
C	S	R	S	S	S	R	S	28.57
CIP	R	R	R	R	R	R	I	85.71
NOR	R	R	R	R	R	R	S	85.71
CL	R	R	R	R	R	R	R	100
CTX	R	R	R	R	R	I	S	71.43
FOX	R	R	R	R	I	S	I	57.14
AK	R	R	R	R	S	R	I	71.43
AMP	R	R	R	R	R	R	R	100
CAR	R	R	R	R	R	R	R	100
E	R	R	R	R	R	R	R	100
TE	R	R	R	R	R	R	R	100
DO	R	R	R	R	R	R	R	100
F	R	R	R	R	R	R	R	100
ATM	R	R	R	R	R	R	I	85.71
MEM	R	R	R	S	R	S	S	57.14
IPM	R	R	R	R	R	S	S	71.43
PB	R	S	R	I	I	I	I	28.57
RD	R	R	R	R	R	R	R	100
Amount	17	18	18	16	15	15	8	

R resistant. S susceptible. I intermediate. SXT sulfamethoxazole and trimethoprim. FFC florfenicol. C chloramphenicol. CIP ciprofloxacin. NOR norfloxacin. CL cephalexin. CTX cefotaxime. FOX cefoxitin. AK amikacin. AMP ampicillin. CAR carbenicillin. E erythromycin. TE tetracycline. DO doxycycline. F nitrofurantoin. ATM aztreonam. MEM meropenem. IPM imipenem. PB polymyxin B. RD rifampin.

**Table 6:** Distribution of virulence genes of *K. pneumoniae* isolates from avian.

Virulence Gene	Strain						
	*KP*820	*KP*826	*KP*911	*KP*926	*KP*1016	*KP*1103	*KP*1116
*uge*	+	+	+	+	+	+	+
*wabG*	+	+	+	+	+	+	+
*rmpA*	−	−	−	−	−	−	−
*rmpA2*	−	−	−	−	−	−	−
*magA*	−	−	−	−	−	−	−
*K2*	−	−	−	−	−	−	−
*wcaG*	−	−	−	−	−	−	−
*fimH*	+	+	+	+	+	+	+
*mrkD*	+	+	+	+	+	+	+
*allS*	−	−	−	−	−	−	−
*ureA*	+	+	+	+	+	+	+
*entB*	+	+	+	+	+	+	+
*iutA*	−	−	−	−	−	−	−
*iucA*	−	−	−	−	−	−	−
*iroB*	−	−	−	−	−	−	−
*ybtS*	−	−	−	−	−	−	−
*irp2*	−	−	−	−	−	−	−
*fyuA*	−	−	−	−	−	−	−
*aerobactin*	−	−	−	−	−	−	−
*kfu*	+	−	−	+	−	+	−
*peg-344*	−	−	−	−	−	−	−
Amount	7	6	6	7	6	7	6

+ Positive. − Negative.

**Table 7:** Distribution of virulence genes of *K. pneumoniae* isolates from humans.

Virulence Gene	Strain						
	*KP*33	*KP*34	*KP*35	*KP*36	*KP*37	*KP*15	*KP*20
*uge*	+	+	+	+	+	+	+
*wabG*	+	+	+	+	+	+	+
*rmpA*	+	−	−	−	−	−	+
*rmpA2*	+	+	−	−	−	−	+
*magA*	−	−	−	−	−	−	+
*K2*	−	−	−	−	−	+	−
*wcaG*	−	−	−	−	−	−	+
*fimH*	+	+	+	+	+	+	+
*mrkD*	+	+	+	+	+	+	+
*allS*	−	−	−	−	−	−	+
*ureA*	+	+	+	+	+	+	+
*entB*	+	+	+	+	+	+	+
*iutA*	+	+	−	−	−	−	+
*iucA*	+	+	−	−	−	−	+
*iroB*	−	−	−	−	−	+	+
*ybtS*	+	+	+	+	+	+	+
*irp2*	+	+	+	+	+	+	+
*fyuA*	+	+	+	+	+	+	+
*aerobactin*	−	−	−	−	−	+	+
*kfu*	−	−	+	+	+	−	+
*peg-344*	+	−	−	−	−	−	+
Amount	14	12	10	10	10	12	20

+ Positive. − Negative.

**Table 8:** Spearman correlation analysis.

Variate	The Number of Virulence Genes	The LD_50_ of the *Galleria mellonella*	The LD_50_ of Mice
The number of virulence genes	1		
The LD_50_ of the *Galleria mellonella*	−0.799 **	1	
The LD_50_ of mice	−0.87 **		1

**, *p* < 0.01.

## Data Availability

The data are contained within this article.

## Supplementary Materials

The following supporting information can be downloaded at: https://www.mdpi.com/article/10.3390/vetsci12070628/s1, Table S1: Primers used in this study; Figure S1: The number of virulence genes carried by avian and human *K. pneumoniae*. **, *p* < 0.01; Figure S2: The adhesion and invasion rate of Calu-3 cells by avian and human *Klebsiella pneumoniae*. *, *p* < 0.05, **, *p* < 0.01; Figure S3: The phagocytic rate of macrophages to avian strains and human strains. **, *p* < 0.01; Figure S4: (A)–(L) Survival rate of *Galleria mellonella* (*n* = 10) infected with *K. pneumoniae* isolates; Figure S5: (A)–(L) The body weight changes of mice (*n* = 5) intratracheally injected with *K. pneumoniae* isolates; Figure S6: (A)–(L) Survival rate of mice (*n* = 5) intratracheally injected with *K. pneumoniae* isolates.
