# Supplementary material for: Biofilm Formation, Antibiotic Resistance, and Virulence Analysis of Human and Avian Origin Klebsiella pneumoniae from Jiangsu, China
Source: Vet Sci. 2025 Jun 30;12(7):628. doi: 10.3390/vetsci12070628 (PMC12299882; doi:10.3390/vetsci12070628)
Supplement: Supplementary file 1 [file vetsci-12-00628-s001.zip › vetsci-3638944-supplementary.pdf]

**Table S1.** Primers used in this study.

| Primer name | DNA Sequence (5' to 3') | Amplicon size (bp) |
|---|---|---|
| 16S-F | AGAGTTTGATCCTGGCTCAG | 1600 |
| 16S-R | GGTTACCTTGTTACGACTT | |
| *rpoB*-F | GGCGAAATGGCWGAGAACCA | 1080 |
| *rpoB*-R | GAGTCTTCGAAGTTGTAACC | |
| *gapA*-F | TGAAATATGACTCCACTCACGG | 663 |
| *gapA*-R | CTTCAGAAGCGGCTTTGATGGCTT | |
| *mdh*-F | CCCAACTCGCTTCAGGTTCAG | 514 |
| *mdh*-R | CCGTTTTTCCCCAGCAGCAG | |
| *pgi*-F | GAGAAAAACCTGCCTGTACTGCTGGC | 718 |
| *pgi*-R | CGCGCCACGCTTTATAGCGGTTAAT | |
| *phoE*-F | ACCTACCGCAACACCGACTTCTTCGG | 600 |
| *phoE*-R | TGATCAGAACTGGTAGGTGAT | |
| *infB*-F | CTCGCTGCTGGACTATATTCG | 463 |
| *infB*-R | CGCTTTCAGCTCAAGAACTTC | |
| *tonB*-F | CTTTATACCTCGGTACATCAGGTT | 541 |
| *tonB*-R | ATTCGCCGGCTGRGCRGAGAG | |
| *uge*-F | TCTTCACGCCTTCCTTCACT | 534 |
| *uge*-R | GATCATCCGGTCTCCCTGT | |
| *wabG*-F | ACCATCGGCCATTTGATAGA | 683 |
| *wabG*-R | CGGACTGGCAGATCCATATC | |
| *rmpA*-F | CATAAGAGTATTGGTTGACAG | 461 |
| *rmpA*-R | CTTGCATGAGCCATCTTTCA | |
| *rmpA2*-F | GTGCAATAAGGATGTTACATTA | 430 |
| *rmpA2*-R | GGATGCCCTCCTCCTG | |
| *magA*-F | GGTGCTCTTTACATCATTGC | 1283 |
| *magA*-R | GCAATGGCCATTTGCGTTAG | |
| *K2*-F | CAACCATGGTGGTCGATTAG | 531 |
| *K2*-R | TGGTAGCCATATCCCTTTGG | |
| *wcaG*-F | GGTTGGKTCAGCAATCGTA | 169 |
| *wcaG*-R | ACTATTCCGCCAACTTTTGC | |
| *fimH*-F | TGCTGCTGGGCTGGTCGATG | 688 |
| *fimH*-R | GGGAGGGTGACGGTGACATC | |
| *mrkD*-F | AAGCTATCGCTGTACTTCCGGCA | 340 |
| *mrkD*-R | GGCGTTGGCGCTCAGATAGG | |
| *allS*-F | CATTACGCACCTTTGTCAGC | 764 |
| *allS*-R | GAATGTGTCGGCGATCAGCTT | |
| *ureA*-F | GCTGACTTAAGAGAACGTTATG | 337 |
| *ureA*-R | GATCATGGCGCTACCT(C/T)A | |
| *entB*-F | GTCAACTGGGCCTTTGAGCCGTC | 400 |
| *entB*-R | TATGGGCGTAAACGCCGGTGAT | |
| *iutA*-F | GGGAAAGGCTTCTCTGCCAT | 920 |
| *iutA*-R | TTATTCGCCACCACGCTCTT | |
| *iucA*-F | AATCAATGGCTATTCCCGCTG | 239 |
| *iucA*-R | CGCTTCACTTCTTTCACTGACAGG | |
| *iroB*-F | ATCTCATCATCTACCCTCCGCTC | 235 |
| *iroB*-R | GGTTCGCCGTCGTTTTCAA | |
| *ybtS*-F | GACGGAAACAGCACGGTAAA | 242 |
| *ybtS*-R | GAGCATAATAAGGCGAAAGA | |

| | | |
|---|---|---|
| *irp2*-F | GCTACAATGGGACAGCAACGAC | 230 |
| *irp2*-R | GCAGAGCGATACGGAAAATGC | |
| *fyuA*-F | TTTCCACCAACACCATCCAG | 817 |
| *fyuA*-R | CAGGTCAGGTCACTGTATGC | |
| *aerobactin*-F | GCATAGGCGGATACGAACAT | 556 |
| *aerobactin*-R | CACAGGGCAATTGCTTACCT | |
| *kfu*-F | CAACCATGGTGGTCGATTAG | 638 |
| *kfu*-R | GGGTCTGGCGCAGAGTATGC | |
| *peg-344*-F | CTTGAAACTATCCCTCCAGTC | 508 |
| *peg-344*-R | CCAGCGAAAGAATAACCCC | |

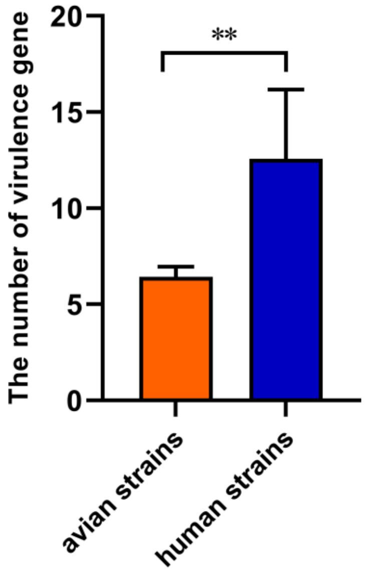


**Figure S1.** The number of virulence genes carried by avian and human *K. p*neumoniae. **, $p < 0.01$.

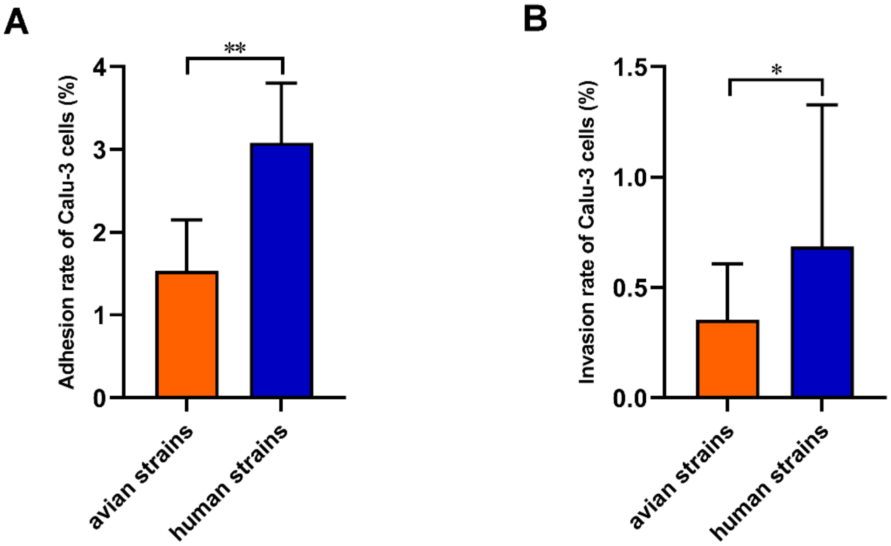


**Figure S2.** The adhesion and invasion rate of Calu-3 cells by avian and human *Klebsiella pneumoniae*. *, $p < 0.05$, **, $p < 0.01$.

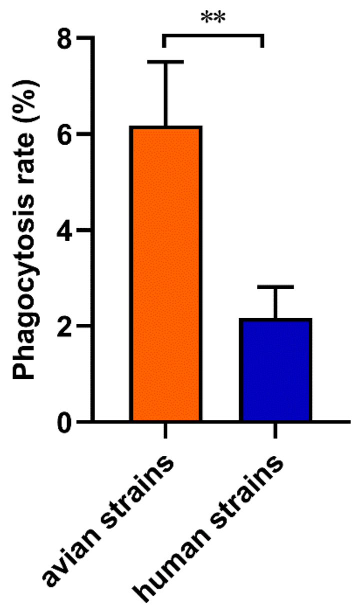


**Figure S3.** The phagocytic rate of macrophages to avian strains and human strains. **, $p < 0.01$.

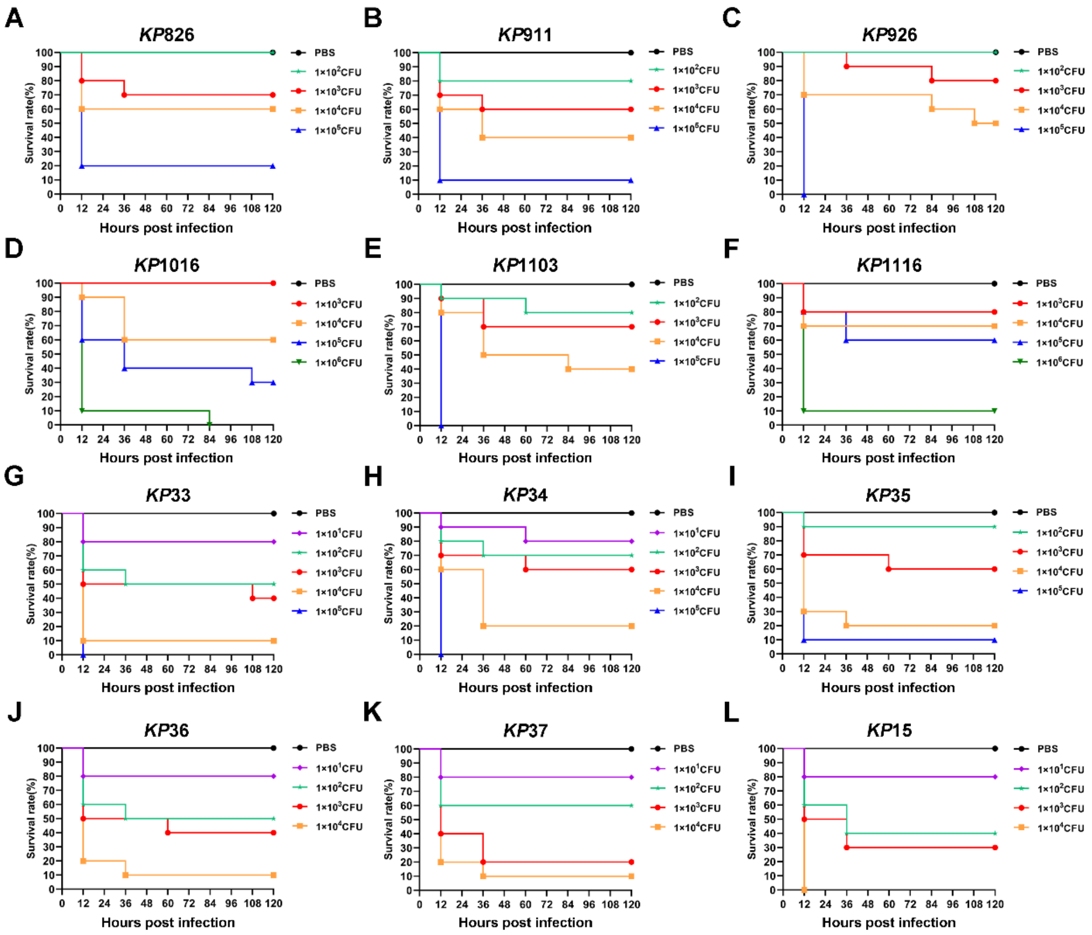


**Figure S4.** (A)-(L) Survival rate of *Galleria mellonella* ($n$=10) infected with *K. pneumoniae* isolates.

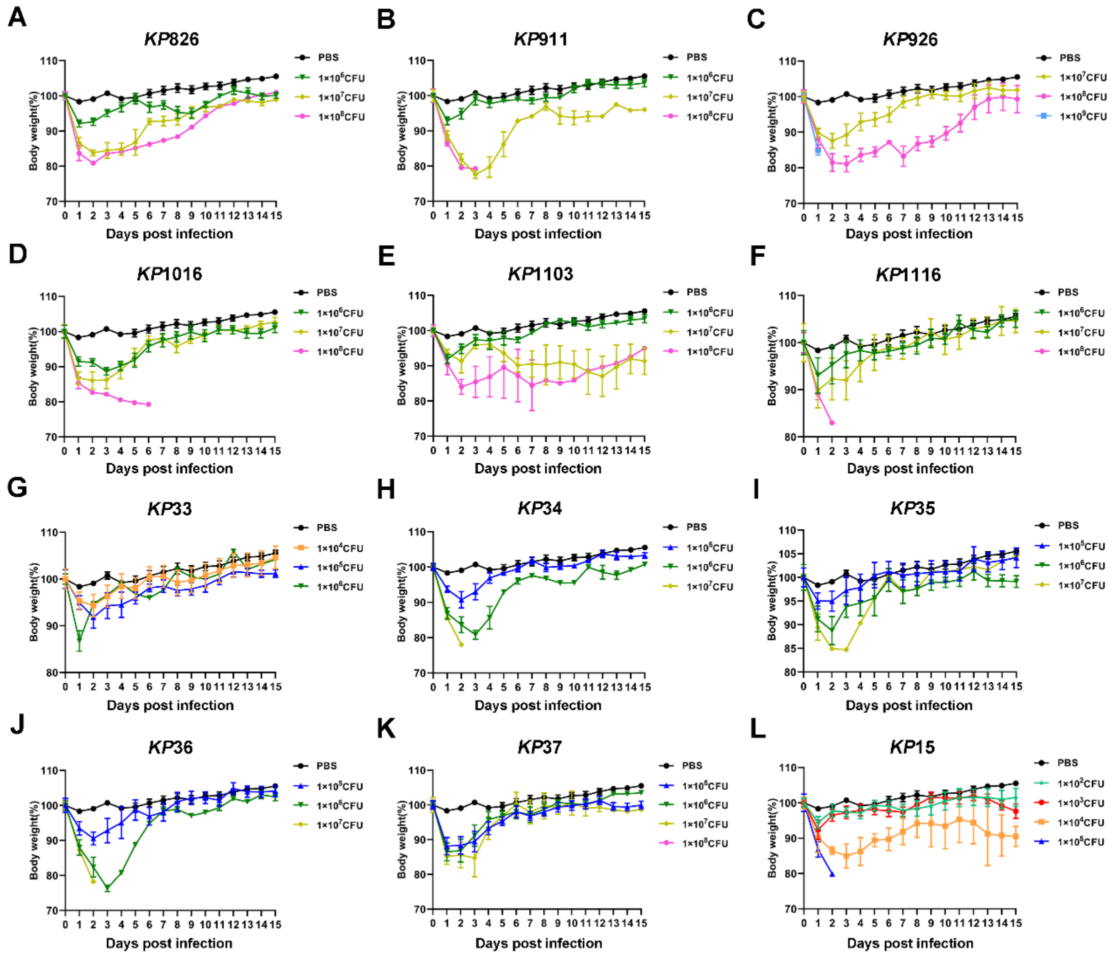

**Figure S5.** (A)-(L) The body weight changes of mice ($n$=5) intratracheally injected with *K. pneumoniae* isolates.

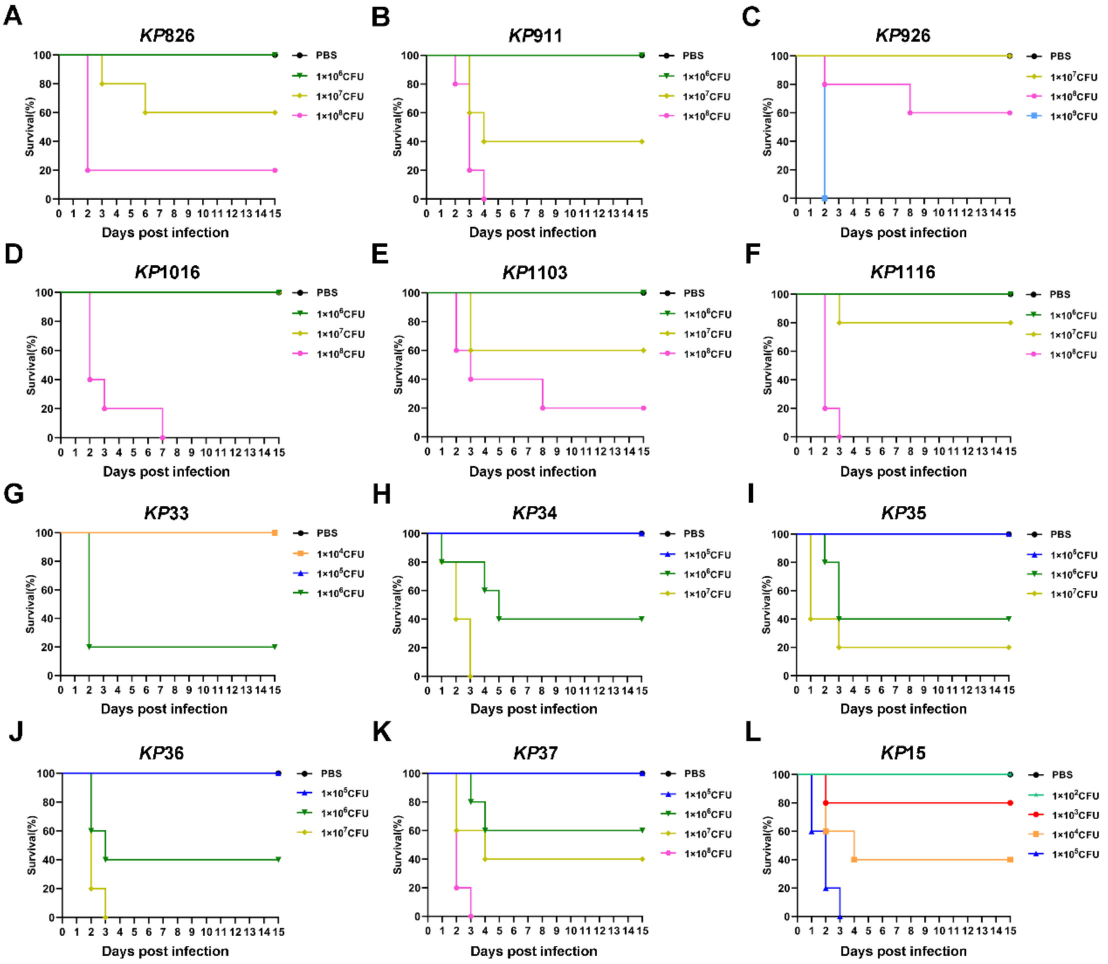


**Figure S6.** (A)-(L) Survival rate of mice (*n*=5) intratracheally injected with *K. pneumoniae* isolates.
